# The Prevalence of Tobacco, Heated Tobacco, and E-Cigarette Use in Poland: A 2022 Web-Based Cross-Sectional Survey

**DOI:** 10.3390/ijerph19084904

**Published:** 2022-04-18

**Authors:** Mateusz Jankowski, Aurelia Ostrowska, Radosław Sierpiński, Adam Skowron, Janusz Sytnik-Czetwertyński, Wojciech Giermaziak, Mariusz Gujski, Waldemar Wierzba, Jarosław Pinkas

**Affiliations:** 1 School of Public Health, Centre of Postgraduate Medical Education, 01-826 Warsaw, Poland; aurelia.ostrowska@cmkp.edu.pl (A.O.); adam.skowron@cmkp.edu.pl (A.S.); janusz.sytnik-czetwertynski@cmkp.edu.pl (J.S.-C.); jpinkas@cmkp.edu.pl (J.P.); 2 Faculty of Medicine, Collegium Medicum, Cardinal Stefan Wyszyński University, 01-815 Warsaw, Poland; radoslaw.sierpinski@abm.gov.pl; 3 The Stanisław Konopka Main Medical Library, 00-791 Warsaw, Poland; w.giermaziak@gbl.waw.pl; 4 Department of Public Health, Medical University of Warsaw, 02-097 Warsaw, Poland; mariusz.gujski@wum.edu.pl; 5 UHE Satellite Campus in Warsaw, University of Humanities and Economics in Łódź, 01-513 Warsaw, Poland; waldemar.wierzba@cskmswia.gov.pl; 6 Central Clinical Hospital of the Ministry of the Interior and Administration in Warsaw, 02-507 Warsaw, Poland

**Keywords:** tobacco, smoking, cigarettes, e-cigarettes, heated tobacco, prevalence, epidemiology, Poland

## Abstract

Monitoring tobacco use on a regular schedule is a basic tool of tobacco control policy. This study aimed (1) to assess the current prevalence and patterns of tobacco and e-cigarette use, as well as (2) to identify socioeconomic factors associated with smoking behavior among adults in Poland. This cross-sectional study was carried out in March 2022 on a nationwide, representative sample of 1090 adults in Poland. The computer-assisted web interview (CAWI) technique was used. Daily tobacco smoking was declared by 28.8% of respondents (27.1% of females and 30.8% of males; *p* = 0.2) and 4.2% were occasional smokers (4.2% of females and 4.3% of males; *p* = 0.8). Most of the current smokers (62.1%) smoked regular cigarettes and 25.2% smoked hand-rolled cigarettes. The prevalence of daily e-cigarette use was 4.8% (4.0% among females and 5.6% among males; *p* = 0.2). Daily heated tobacco use was declared by 4.0% of respondents (5.1% of females and 2.9% of males; *p* = 0.07). Age, having children, and educational level were significantly associated with current daily tobacco smoking. This study revealed a high prevalence of tobacco and e-cigarette use among adults in Poland. The presented data underscore the importance of further improvements in adopting a comprehensive tobacco control strategy in Poland.

## 1. Introduction

Tobacco use, primarily cigarette smoking, is the leading cause of preventable disease and death [1,2]. Smoking is a known cause of cancer, heart disease, stroke, lung diseases, diabetes, and chronic obstructive pulmonary disease (COPD) [3,4]. It is estimated that tobacco kills more than 8 million people globally each year [4]. Tobacco-related disease costs (from health expenditures and productivity losses together) the global economy over 1 trillion US dollars per year, which is approximately 1.8% of the global gross domestic product (GDP) [5].

The World Health Organization (WHO) Framework Convention on Tobacco Control (FCTC) requires member states to consistently collect national data on tobacco use [6]. In 2008, the WHO launched the MPOWER policy package—a basic tool that helps countries reduce the demand for tobacco [7,8]. The first point of the six MPOWER technical measures involves “monitoring tobacco use and prevention policies” [7]. Regular monitoring of tobacco use allows for the evaluation of tobacco control activities, as well as for the identification of specific risk groups and changes in smoking behaviors in society [7,8]. 

At an international level, the prevalence of tobacco use in Europe is estimated based on the findings from the WHO Global Adult Tobacco Survey (GATS) [9] and the European Commission Special Eurobarometer [10,11]. 

According to the WHO estimates, 1.1 billion people globally smoke [4]. Europe is the region with the highest prevalence of tobacco smoking [12]. It is estimated that more than one-quarter of adults in Europe smoke [11,12]. The highest smoking prevalence is observed in Eastern Europe (28%) and the lowest in Northern Europe (20%) [13]. Moreover, it is estimated that approximately 2–2.5% of Europeans regularly use electronic cigarettes (e-cigarettes) [11,14]. Moreover, heated tobacco products (such as IQOS or glo) are gaining popularity [15]. 

According to the Special Eurobarometer 506, the proportion of smokers in the EU and UK has decreased from 26% in 2017 [10] to 23% in 2020 [11]. In the EU, the highest prevalence of smoking was observed in Greece (42%), Bulgaria (38%), and Croatia (36%), whereas the lowest proportion of smokers was observed in the Netherlands (12%) and Sweden (7%) [11]. Between 2017 and 2020, the prevalence of e-cigarette use remained stable—both 2% in 2017 [10] and 2020 [11]. In 2020, 1% of EU citizens were current heated tobacco users [11]. 

Poland is a European country with a significant burden of tobacco-related diseases and deaths [16,17]. In Poland, smoking contributes to over 70,000 deaths every year [17]. After introducing wide-ranging tobacco control measures in the 1990s, Poland experienced a steady decline in cigarette consumption and smoking prevalence [17]. However, in 2020, still, 26% of Poles aged 15 and over were smokers [11]. The prevalence of smoking, as well as the burden of tobacco-related diseases in Poland, is higher among males than females [16,18]. In Poland, the proportion of smokers is especially high among socially disadvantaged populations [19]. Moreover, growing evidence suggests that e-cigarettes and heated tobacco products are gaining popularity in Poland [15,20,21]. 

The COVID-19 pandemic has led to sudden changes in daily life and to modifications in health behaviors [22]. The stress and anxiety experienced during the pandemic may have led to an increase in tobacco use [22,23]. Lockdown, remote work, and remote learning may also have had an impact on lifestyle and smoking behaviors [22,23,24]. Data on dietary choices and habits during the COVID-19 lockdown show that 45% of smokers experienced a rise in smoking frequency during the lockdown [24]. However, there are a lack of nationwide representative epidemiological data on tobacco and e-cigarette use in Poland during the COVID-19 pandemic. 

This study aimed (1) to assess the current prevalence and patterns of tobacco and e-cigarette use, as well as (2) to identify socioeconomic factors associated with smoking behaviors among adults in Poland.

## 2. Materials and Methods

### 2.1. Study Design and Population 

This cross-sectional study was carried out between 4 and 7 March 2022, on a representative nationwide sample of 1090 individuals aged 18 years and older in Poland. The computer-assisted web interview (CAWI) technique was used [25]. All of the interviews were carried out by a specialized survey company (Ogólnopolski Panel Badawczy Sp. z o.o., Warszawa, Poland) [26] on behalf of the research team, which provided the scientific context of the survey. 

Respondents were selected from Ogólnopolski Panel Badawczy Sp. z o.o. as a part of the Omnibus survey [26]. The operational number of the Ogólnopolski Panel Badawczy Sp. z o.o. is over 110,000 registered and verified individuals aged 15 years and older, and is actively updated to maintain representativeness for the Polish population. Data collection through the Ogólnopolski Panel Badawczy Sp. z o.o. methodology (using a dedicated IT system) has been used in previously published papers [27,28,29].

A non-probability quota sampling was applied [26]. Respondents were selected based on the stratification model, including gender, age, size of domicile, and the territorial distribution within 16 administrative regions in Poland. The stratification was based on demographic data from the Central Statistical Office, Warsaw, Poland [30].

Participation in the study was voluntary and anonymous. All participants provided their informed consent. The study protocol was approved by the Ethical Review Board at the Centre of Postgraduate Medical Education, Warsaw, Poland (consent number 21/2022; date of approval: 16 February 2022).

### 2.2. Questionnaire and Study Measures

The research tool was a questionnaire developed for the purpose of this study. In preparation for the questionnaire, we analyzed the previously published nationwide cross-sectional surveys on tobacco use, with particular emphasis on the Global Adult Tobacco Survey (GATS) [9]. The questionnaire included 12 questions on tobacco products, heated tobacco products, and e-cigarette use. Questions also addressed sociodemographic characteristics.

Smoking status: Respondents were asked about their smoking status, using the questions, “Have you ever smoked at least 100 cigarettes (or a similar amount of other tobacco products e.g., pipes, cigars, cigarillos) in your lifetime?” and “Do you currently smoke?”. Current tobacco smokers were respondents who reported having smoked ≥100 cigarettes (or a similar amount of other tobacco products) during their lifetime and who currently smoke. Moreover, based on the answer to the question, “During the past six months, have you smoked tobacco daily?”, this group was divided into “daily” smokers or “occasional” smokers. Current tobacco smokers were also asked about the type of tobacco products that they smoke (regular cigarettes, menthol cigarettes, slim cigarettes, hand-rolled cigarettes, cigars, cigarillos, pipe, or shisha). Respondents who reported having smoked ≥100 cigarettes (or other tobacco products) during their lifetime but were not smoking at the time of the study were classified as former smokers. Non-smokers were respondents who reported having smoked fever than 100 cigarettes (or other tobacco products) during their lifetime and who do not smoke now.

The mean number of cigarettes or other tobacco products smoked per day was calculated among daily smokers. Current e-cigarette use was defined based on the answers to the question, “Do you currently use an e-cigarette (daily)?”. Current heated tobacco use was defined based on the answers to the question, “Do you currently use heated tobacco products, e.g., IQOS or glo, (daily)?”.

Sociodemographics: Questions related to sociodemographic data included the following: gender (male/female), age (years), educational level (primary, vocational, secondary, or higher), having children, marital status (single, married, informal relationship, divorced, or widowed), occupational status, financial situation, and place of residence. The occupational activity was classified as active (currently employed) or passive (currently unemployed). The financial situation was assessed with the question, “How do you assess your own/your family’s financial situation? (very good/good/rather good, moderate, bad/rather bad/very bad)” (this question was chosen due to the numerous missing data in the case of the question about the amount of monthly income). 

### 2.3. Statistical Analysis

The data were analyzed with SPSS version 28 (IBM, Armonk, NY, USA). The normality of distributions of continuous variables was assessed by the Shapiro−Wilk test. Statistical significance of the differences between continuous variables was analyzed by the independent samples t-test or, if the assumptions for this were not met, the Mann−Whitney U test was used. The distribution of categorical variables was shown by frequencies and proportions. Statistical testing to compare categorical variables was completed using the independent samples chi-square test. In the case of less than five subjects, the Fisher exact test was used for 2 × 2 tables, and in the case of more categories, the Fisher−Freeman−Halton exact test was used.

Associations between personal characteristics (gender, age, marital status, having children, place of residence, educational level, occupational status, and financial situation) with smoking status were conducted using logistic regression analyses. Daily smoking was considered as a dependent variable in the model. The socio-demographic characteristics (gender, age, marital status, having children, place of residence, educational level, occupational status, and financial situation) were considered as independent variables. In the univariate logistic regression analyses, all variables were considered separately. Multivariable logistic regression analyses included all the variables significantly associated with daily cigarette smoking in any of the univariate models (*p* < 0.05) 

The strength of association was measured by the odds ratio (OR) and 95% confidence intervals (CI). Statistical inference was based on the criterion *p* < 0.05.

## 3. Results

### 3.1. Characteristics of the Study Population

The analysis was based on responses to survey forms received from 1090 individuals (52.6% females), with a mean age of 45.2 ± 16.2 (18–84) years. The characteristic of the sample classified by smoking status, separately for men and women, is presented in Table 1. 

The prevalence of smoking was 28.8% (27.1% of females and 30.8% of males; *p* = 0.2). Current daily smoking was declared by 22.9% of females and 26.5% of males (*p* = 0.2). Moreover, 4.2% of females and 4.3% of males were current occasional smokers (*p* = 0.8). Among the females, there were no significant differences in the prevalence of smoking by socioeconomic factors (Table 1).

Among the males, there were significant differences in the prevalence of smoking by marital status, having children, educational level, occupational status, as well as financial status (*p* < 0.05). Details are presented in Table 1.

### 3.2. Smoking Patterns

Most of the current smokers (62.1%) smoked regular cigarettes and one-quarter (25.2%) smoked hand-rolled cigarettes (Table 2). Males more often smoked hand-rolled cigarettes than females (34.6% vs. 15.5%, respectively; *p* < 0.001). Moreover, almost one-quarter of current smokers smoked slim cigarettes (30.3% of females and 18.9% of males; *p* = 0.02). Heated tobacco products were used by 16.8% of the current smokers. Approximately 5% of current smokers smoked cigars, cigarillos, or pipe, wherein males compared to females more often (*p* < 0.05) declared the use of these tobacco products (Table 2). Current smokers smoked an average of 12 regular cigarettes a day, without significant differences (*p* > 0.05) by gender. Those smokers who smoked hand-rolled cigarettes smoked an average of 13.3 hand-rolled cigarettes a day. Details are presented in Table 2. 

The overall prevalence of dual use was 4.4% (*n* = 48), wherein 2.0% (*n* = 22) were dual cigarette/e-cigarette users, 2.2% (*n* = 24) were dual cigarette/heated tobacco users, and 0.2% (*n* = 2) were dual e-cigarette/heated tobacco users. Moreover, 1% (*n* = 11) were triple users (daily tobacco, e-cigarette, and heated tobacco use). 

### 3.3. Associates of Smoking Status

The results of the univariate and multivariable logistic regression analyses are presented in Table 3. Age, having children, and educational level were significantly associated with current daily smoking among adults in Poland (Table 3). Participants aged 50–59 years (OR = 1.60; 95% CI:1.05–2.43; *p* < 0.05), those who had children (OR = 2.08; 95% CI:1.31–3.31; *p* < 0.01), and respondents without a higher education (OR = 2.00; 95% CI:1.46–2.73; *p* < 0.001) had higher odds of being current tobacco smokers.

### 3.4. E-Cigarette Use

The overall prevalence of daily e-cigarette use was 4.8% (4.0% of females and 5.6% of males; *p* = 0.2). There were no significant differences in the prevalence of e-cigarette use according to socioeconomic factors (Table 4). Out of 52 e-cigarette users, 17 were exclusive e-cigarette users (1.6% of the total sample). 

### 3.5. Heated Tobacco Use

The overall prevalence of daily heated tobacco use was 4.0% (5.1% of females and 2.9% of males; *p* = 0.07). There were significant differences in the prevalence of daily heated tobacco use by age (Table 5). Moreover, the prevalence of daily heated tobacco use was almost three times higher among those who were occupationally active compared to those who were occupationally passive (5.5% vs. 1.9%; *p* = 0.003). Out of the 44 heated tobacco users, seven were exclusive heated tobacco users (0.6% of the total sample). 

## 4. Discussion

To the best of our knowledge, this is the most up-to-date study on the prevalence of tobacco, heated tobacco, and e-cigarette use among adults in Poland. This study was carried out two years after the detection of the first COVID-19 case in Poland. This study revealed a high prevalence of tobacco use among adults in Poland. Moreover, we observed a markable percentage of adult Poles who declared daily heated tobacco (4.0%) or e-cigarette (4.8%) use. In this study, there were no gender differences in tobacco, heated tobacco, or e-cigarette use, which indicates a blurring of differences in smoking behaviors between the sexes. 

Data on tobacco use in Poland are regularly monitored by the Centre for Public Opinion Research, the European Commission (as a part of the Special Eurobarometer Survey), or the Chief Sanitary Inspectorate [10,11,16,31]. In September 2019, the prevalence of smoking among Poles aged 15 and over was 22.3% (19.1% of females (18% daily smokers and 1.1% occasional smokers) and 25.9% (24.4% daily smokers and 1.5% occasional smokers) of males; *p* = 0.01) [16]. According to the Special Eurobarometer 506, in 2020, 26% of Poles aged 15 and over were current smokers [11]. In this study, current tobacco use was declared by 28.8% (24.6% daily and 4.2% occasional smokers) of adults in Poland, without significant differences between sexes. Compared to 2019, we observed changes in smoking behaviors among adults in Poland. In 2022, there were no significant differences in smoking prevalence between males and females. This finding is in line with global trends in tobacco use (decrease in smoking among males and increase in smoking among females) [32]. The tobacco industry targets females in order to increase its consumer base and to replace those consumers who quit or who die prematurely from tobacco-related diseases. Moreover, we did not observe significant differences in tobacco use between rural and urban areas. In previous years (2017 or 2019), those who lived in small or medium-size cities had higher odds of current tobacco use. The lack of differences in tobacco use by place of residence may result from socioeconomic changes in Polish society, reducing unemployment (especially in small towns), and increasing living standards [33].

According to the Special Eurobarometer 506, carried out in 2020, the highest prevalence of tobacco use was observed among those aged 25−54 years old [11]. Moreover, those with primary or vocational education smoked the most. In the EU, smoking prevalence is greater among those who are unemployed [11]. The socio-demographic profile of smokers in the EU did not change between 2017 [10] and 2020 [11]. In this study, the prevalence of tobacco use was the highest among individuals aged 40–59 years, as well as for those without higher education, which is in line with the EU data [11]. 

In line with previously published studies, the most frequently used tobacco product was regular cigarettes [16]. The percentage of smokers who used hand-rolled cigarettes increased from 21.6% in 2019 to 25.2% in 2022 [16]. This increase may result from the fact that in Poland, loose tobacco is subject to a lower tax than regular cigarettes, which makes hand-rolled cigarettes cheaper than regular cigarettes. This issue may be particularly important in the case of increasing tobacco taxation in Poland. Moreover, we observed a marked proportion of smokers who used slim cigarettes (24.5%). Cigarette packs can influence perceptions of appeal, harm, and taste [34]. Cigarette appeal is especially important for females [35]. This phenomenon may impact gender differences in the prevalence of slim cigarettes (with “cooler” and attractive packaging) and hand-rolled cigarettes (without attractive packing) use.

In 2020, 2% of Poles aged 15 and over declared current e-cigarette use [11]. In this study, 4.8% of adult Poles were daily e-cigarette users. This finding suggests a markable increase in e-cigarette use in Poland and requires further investigation. The potential impact of the COVID-19 pandemic and lockdown on smoking behaviors should be also considered, both among adolescents (particularly vulnerable groups) and among adults.

In 2017, the first heated tobacco products were introduced to the Polish market [21]. These products are marketed through social media, dedicated brand ambassadors, as well as dedicated stands in shopping malls [15]. Due to the higher price than traditional cigarettes, heated tobacco products are mainly marketed to occupationally active adults from larger cities [15]. Data from Japan—one of the first heated tobacco markets—showed that between 2015 and 2017, the proportion of current heated tobacco users increased from 0.3% to 3.6% [36]. A similar trend was observed in our study. Between 2019 and 2022, the prevalence of heated tobacco users increased from 0.4% to 4.0% [16]. Moreover, this study confirmed that adults aged 30–49 years, as well as those who lived in the largest cities (above 500,000 residents), had higher odds of daily heated tobacco use. The availability of different nicotine-containing products that are targeted and marketed to different social groups points to an urgent need to provide educational campaigns on different nicotine products (including e-cigarettes and heated tobacco products) and their health consequences. 

There are several practical implications of this study. The findings from this study suggest that there is an urgent need to conduct a nationwide anti-tobacco campaign that will also address the use of e-cigarettes and heated tobacco products. Moreover, the gender gap in tobacco use has decreased. This finding indicates changes in social attitudes towards tobacco and the growing problem of tobacco use among women. Moreover, the proportion of adults who are using e-cigarettes or heated tobacco products is increasing. The growing popularity of e-cigarettes and heated tobacco products, as well as dual or triple use points to the urgent need to adjust tobacco control laws to new trends in nicotine product use. Moreover, further national studies are needed to confirm the findings on increase in the prevalence of e-cigarette and heated tobacco products use.

This study has several limitations. This study was carried out using the computer-assisted web interviewing (CAWI) research method, which is limited to internet users (nevertheless, more than 90% of households in Poland now have internet access) [37]. Data were collected as a part of the Omnibus survey, so the potential impact of other items included in the overall survey on the responses to questions about smoking behaviors is unknown. Moreover, the potential risk of non-response bias cannot be excluded. Nevertheless, the same methods were used in previously published data aimed at a similar public health area [27,28,29]. In this study, smoking status was defined based on the self-reported data on tobacco use, so we cannot exclude the possibility of recall bias. The biomarkers of tobacco smoking [38] were not verified in this study.

## 5. Conclusions

This is the most up-to-date epidemiological study on the prevalence of tobacco and e-cigarette use in Poland. Compared to data from 2019, this study showed a markable increase in the prevalence of smoking both for men and women. Moreover, the current study indicated the blurring of differences in smoking behaviors between the sexes. The prevalence of heated tobacco use increased ten-fold, which points to the emerging tobacco control problem arising from the use of these products. The presented data underscore the importance of further improvements in adopting a comprehensive tobacco control strategy in Poland.

## Figures and Tables

**Table 1:** Characteristics of the study population by smoking status (n = 1090).

Variable	Total Sample	Women			*p*	Men			*p*
		Total	Smokers	Non-Smokers		Total	Smokers	Non-Smokers	
Overall	*n* = 1090 *n* (%)	*n* = 573 *n* (%)	*n* = 155 (27.1%) *n* (%)	*n* = 418 (72.9%) *n* (%)		*n* = 517 *n* (%)	*n* = 159 (30.8%) *n* (%)	*n* = 358 (69.2%) *n* (%)	
Age (years)									
18–29	222 (20.3)	101 (17.6)	24 (23.8)	77 (76.2)	*0.6*	121 (23.4)	35 (28.9)	86 (71.1)	0.06
30–39	231 (21.2)	121 (21.1)	31 (25.6)	90 (74.4)		110 (21.3)	38 (34.5)	72 (65.5)	
40–49	186 (17.1)	96 (16.8)	32 (33.3)	64 (66.7)		90 (17.4)	32 (35.6)	58 (64.4)	
50–59	196 (18.0)	116 (20.2)	32 (27.6)	84 (72.4)		80 (15.5)	30 (37.5)	50 (62.5)	
60+	255 (23.4)	139 (24.3)	36 (25.9)	103 (74.1)		116 (22.4)	24 (20.7)	92 (79.3)	
Marital status									
single	246 (22.6)	105 (18.3)	22 (21.0)	83 (79.0)	0.2	141 (27.3)	31 (22.0)	110 (78.0)	**0.01**
married	555 (50.9)	288 (50.3)	77 (26.7)	211 (73.3)		267 (51.6)	81 (30.3)	186 (69.7)	
informal relationship	162 (14.9)	89 (15.5)	32 (36.0)	57 (64.0)		73 (14.1)	32 (43.8)	41 (56.2)	
divorced	58 (5.3)	45 (7.9)	13 (28.9)	32 (71.1)		13 (2.5)	6 (46.2)	7 (53.8)	
widowed	69 (6.3)	46 (8.0)	11 (23.9)	35 (76.1)		23 (4.4)	9 (39.1)	14 (60.9)	
Having children									
yes	707 (64.9)	393 (68.6)	115 (29.3)	278 (70.7)	0.08	314 (60.7)	109 (34.7)	205 (65.3)	**0.02**
no	383 (35.1)	180 (31.4)	40 (22.2)	140 (77.8)		203 (39.3)	50 (24.6)	153 (75.4)	
Place of residence									
rural	339 (31.1)	174 (30.4)	43 (24.7)	131 (75.3)	0.2	165 (31.9)	47 (28.5)	118 (71.5)	0.5
city up to 20,000 residents	138 (12.7)	75 (13.1)	23 (30.7)	52 (69.3)		63 (12.2)	19 (30.2)	44 (69.8)	
city between 20,000–99,999 residents	253 (23.2)	129 (22.5)	36 (27.9)	93 (72.1)		124 (24.0)	46 (37.1)	78 (62.9)	
city between 100,000–500,000 residents	211 (19.4)	111 (19.4)	37 (33.3)	74 (66.7)		100 (19.3)	28 (28.0)	72 (72.0)	
city above 500,000 residents	149 (13.7)	84 (14.7)	16 (19.0)	68 (81.0)		65 (12.6)	19 (29.2)	46 (70.8)	
Educational level									
primary	22 (2.0)	9 (1.6)	3 (33.3)	6 (66.6)	0.3	13 (2.5)	3 (23.1)	10 (76.9)	**<0.001**
vocational	111 (10.2)	50 (8.7)	14 (28.0)	36 (72.0)		61 (11.8)	24 (39.3)	37 (60.7)	
secondary	507 (46.5)	286 (49.9)	86 (30.1)	200 (69.9)		221 (42.7)	85 (38.5)	136 (61.5)	
higher	450 (41.3)	228 (39.8)	52 (22.8)	176 (77.2)		222 (42.9)	47 (21.2)	175 (78.8)	
Occupational status									
active	659 (60.5)	313 (54.6)	89 (28.4)	224 (71.6)	0.4	346 (66.9)	121 (35.0)	225 (65.0)	**0.003**
passive	431 (39.5)	260 (45.4)	66 (25.4)	194 (74.6)		171 (33.1)	38 (22.2)	133 (77.8)	
Financial situation									
good	455 (41.7)	215 (37.5)	65 (30.2)	150 (69.8)	0.3	240 (46.4)	66 (27.5)	174 (72.5)	**0.02**
moderate	424 (38.9)	244 (42.6)	59 (24.2)	185 (75.8)		180 (34.8)	52 (28.9)	128 (71.1)	
bad	211 (19.4)	114 (19.9)	31 (27.2)	83 (72.8)		97 (18.8)	41 (42.3)	56 (57.7)	

Statistically significant values are marked bold.

**Table 2:** Type of tobacco products used by the smokers *n* = 314.

The Type of Tobacco Products Smoked the Most (*n* = 314)							
Type of Tobacco Products	Total		Women		Men		
	*n*	%	*n*	%	*n*	%	*p*
Regular cigarettes	195	62.1	97	62.6	98	61.6	0.9
Menthol cigarettes	40	12.7	17	11.0	23	14.5	0.4
Slim cigarettes	77	24.5	47	30.3	30	18.9	**0.02**
Hand-rolled cigarettes	79	25.2	24	15.5	55	34.6	**<0.001**
Cigars	14	4.5	2	1.3	12	7.5	**0.007**
Cigarillos	17	5.4	4	2.6	13	8.2	**0.03**
Pipe	16	5.1	4	2.6	12	7.5	**0.04**
Shisha	7	2.2	2	1.3	5	3.1	0.3
**Number of cigarettes smoked daily**							
	***n***	**mean (±SD)** **(range)**	***n***	**mean (±SD)** **(range)**	***n***	**mean (±SD)** **(range)**	***p***
Regular cigarettes	195	11.9 ± 11.5 (0–100)	97	10.9 ± 7.3 (0–40)	98	12.9 ± 14.5 (0–100)	0.2
Menthol cigarettes	40	7.0 ± 6.5 (0–25)	17	8.4 ± 7.1 (1–25)	23	6.1 ± 6.0 (0–20)	0.2
Slim cigarettes	77	8.1 ± 6.4 (0–25)	47	8.2 ± 6.1 (0–20)	30	7.9 ± 7.0 (1–25)	0.7
Hand-rolled cigarettes	79	13.3 ± 9.1 (1-40)	24	12.6 ± 10.5 (1–40)	55	13.6 ± 8.6 (1–38)	0.4

SD—standard deviation. Statistically significant values are marked bold.

**Table 3:** Odds ratios (OR) and 95% confidence intervals (CI) for current daily smoking considering selected socioeconomic factors in a representative sample of adults in Poland, *n* = 1090.

Variable	Total (*n*)	Current Daily Smokers		*p*	Univariate Logistic Regression		Multivariable Logistic Regression	
		*n*	%		OR	95% CI	OR	95% CI
Gender								
male	517	137	26.5	0.2	1.22	0.92–1.60		
female	573	131	22.9		1.00	Reference		
Age (years)								
18–29	222	36	16.2	<0.001	0.69	0.43–1.10	0.77	0.42–1.41
30–39	231	56	24.2		1.14	0.75–1.74	1.03	0.63–1.68
40–49	186	61	32.8		1.73 *	1.13–2.66	1.07	0.66–1.73
50–59	196	59	30.1		1.53 *	1.01–2.34	1.60 *	**1.05–2.43**
60+	255	56	22.0		1.00	Reference	1.00	Reference
Marital status								
single	246	43	17.5	0.02	1.00	Reference	1.00	Reference
married	555	138	24.9		1.56 *	1.07–2.29	0.80	0.46–1.35
informal relationship	162	51	31.5		2.17 ***	1.36–3.46	1.46	0.87–2.45
divorced	58	17	29.3		1.96 *	1.02–3.77	1.07	0.49–2.32
widowed	69	19	27.5		1.79	0.96–3.34	0.85	0.41–1.77
Having children								
yes	707	202	28.6	<0.001	1.92 ***	1.41–2.62	2.08 **	**1.31–3.31**
no	383	66	17.2		1.00	Reference	1.00	Reference
Place of residence								
rural	339	80	23.6	0.5	1.23	0.76–1.97		
city up to 20,000 residents	138	33	23.9		1.25	0.71–2.18		
city between 20,000–99,999 residents	253	71	28.1		1.55	0.95–2.51		
city between 100,000–500,000 residents	211	54	25.6		1.36	0.82–2.26		
city above 500,000 residents	149	30	20.1		1.00	Reference		
Higher education								
yes	450	80	17.8	<0.001	1.00	Reference	1.00	Reference
no	640	188	29.4		1.92 ***	1.43–2.59	2.00 ***	**1.46–2.73**
Occupational status								
active	659	176	26.7	0.04	1.34 *	1.01–1.79	1.40	0.99–1.97
passive	431	92	21.3		1.00	Reference	1.00	Reference
Financial situation								
good	455	115	25.3	0.2	1.00	Reference		–
moderate	424	93	21.9		0.83	0.61–1.14		–
bad	211	60	28.4		1.18	0.81–1.69		–

* *p* < 0.05; ** *p* < 0.01; *** *p* < 0.001. Statistically significant values are marked bold.

**Table 4:** The prevalence of e-cigarette use among adults in Poland, *n* = 1090.

Variable	Total (*n*)	Current Daily E-Cigarette Use		*p*
		*n*	%	
Gender				
male	517	29	5.6	0.2
female	573	23	4.0	
Age (years)				
18–29	222	15	6.8	0.1
30–39	231	10	4.3	
40–49	186	12	6.5	
50–59	196	10	5.1	
60+	255	5	2.0	
Marital status				
single	246	8	3.3	0.07
married	555	26	4.7	
informal relationship	162	14	8.6	
divorced	58	3	5.2	
widowed	69	1	1.4	
Having children				
yes	707	36	5.1	0.5
no	383	16	4.2	
Place of residence				
rural	339	9	2.7	0.2
city up to 20,000 residents	138	7	5.1	
city between 20,000–99,999 residents	253	16	6.3	
city between 100,000–500,000 residents	211	14	6.6	
city above 500,000 residents	149	6	4.0	
Higher education				
yes	450	21	4.7	0.9
no	640	31	4.8	
Occupational status				
active	659	36	5.5	0.2
passive	431	16	3.7	
Financial situation				
good	455	20	4.4	0.9
moderate	424	21	5.0	
bad	211	11	5.2	

**Table 5:** The prevalence of heated tobacco use among adults in Poland, *n* = 1090.

Variable	Total (*n*)	Current Daily Heated Tobacco Use		*p*
		*n*	%	
Gender				
male	517	15	2.9	0.07
female	573	29	5.1	
Age (years)				
18–29	222	8	3.6	**0.01**
30–39	231	14	6.1	
40–49	186	13	7.0	
50–59	196	7	3.6	
60+	255	2	0.8	
Marital status				
single	246	11	4.5	0.5
married	555	18	3.2	
informal relationship	162	8	4.9	
divorced	58	2	3.4	
widowed	69	5	7.2	
Having children				
yes	707	29	4.1	0.9
no	383	15	3.9	
Place of residence				
rural	339	9	2.7	0.2
city up to 20,000 residents	138	3	2.2	
city between 20,000–99,999 residents	253	11	4.3	
city between 100,000–500,000 residents	211	11	5.2	
city above 500,000 residents	149	10	6.7	
Higher education				
yes	450	21	4.7	0.4
no	640	23	3.6	
Occupational status				
active	659	36	5.5	**0.003**
passive	431	8	1.9	
Financial situation				
good	455	22	4.8	0.5
moderate	424	14	3.3	
bad	211	8	3.8	

Statistically significant values are marked bold.

## Data Availability

Data are available upon reasonable request. The dataset used to conduct the analyses is available from the corresponding author upon reasonable request.
